# Prognostic value of multiple biomarkers for kidney function decline in type 2 diabetes with relatively preserved kidney function: a systematic review and exploratory meta-analysis

**DOI:** 10.3389/fendo.2026.1938521

**Published:** 2026-10-12

**Authors:** Jie Hu, Xinyu Pan, Shuwu Wei, Junping Wei

**Affiliations:** 1 Department of Endocrinology, Guang’anmen Hospital, China Academy of Chinese Medical Sciences, Beijing, China; 2 Graduate School, Beijing University of Chinese Medicine, Beijing, China

**Keywords:** diabetic kidney disease, type 2 diabetes, biomarkers, TNFR1, TNFR2, KIM-1, meta-analysis

## Abstract

**Background:**

Diabetic kidney disease (DKD) is a dominant driver of chronic kidney disease (CKD) and end-stage renal disease (ESRD). Current risk assessment tools, however, lack adequate sensitivity for early disease detection. This study evaluated the prognostic value of multiple biomarkers for kidney function decline in patients with type 2 diabetes and relatively preserved kidney function.

**Methods:**

This systematic review and meta-analysis followed the Preferred Reporting Items for Systematic Reviews and Meta-Analyses (PRISMA) guidelines. PubMed, EMBASE, Web of Science, and the Cochrane Library were searched from inception to December 31, 2025. Articles were independently screened by two reviewers against pre-specified criteria. Prospective cohort studies reporting associations between baseline biomarkers and kidney function decline were included. Methodological quality was assessed using the Quality In Prognosis Studies (QUIPS) tool. Quantitative pooling was performed only when studies were homogeneous in effect measure and outcome definition. When these criteria were not met, results were presented descriptively.

**Results:**

Nine prospective cohort studies comprising 4,569 participants were included. Circulating TNFR1, TNFR2, and KIM-1 showed potential positive associations with kidney function decline. One study reported an inverse association for circulating Klotho. No significant associations were found for circulating FGF-23. Findings for urinary NGAL and urinary KIM-1 were inconsistent across studies. Due to heterogeneity in effect measures, exposure scales, and outcome definitions, several biomarkers could not be quantitatively pooled. For pooled analyses, each analysis included only two studies; therefore, effect estimates, heterogeneity, and prediction intervals may be unstable, and the results should be considered exploratory.

**Conclusions:**

Current evidence suggests potential associations of circulating TNFR1, TNFR2, and KIM-1 with kidney function decline in type 2 diabetes with relatively preserved kidney function. Given the limited number of studies, these findings are exploratory and require validation in larger prospective studies with standardized assays and outcome definitions.

**Systematic review registration:**

https://www.crd.york.ac.uk/PROSPERO/, identifier CRD420261401816.

## 1 Introduction

Diabetic kidney disease (DKD) has been identified as the most prevalent microvascular complication among patients with type 2 diabetes mellitus (T2DM). Furthermore, it has been recognized as the predominant contributor to the worldwide burden of chronic kidney disease (CKD) and end-stage renal disease (ESRD), thereby presenting a considerable challenge to both clinical management and public health efforts (1, 2). Currently, risk stratification for DKD mainly relies on estimated glomerular filtration rate (eGFR) and the urinary albumin-to-creatinine ratio (UACR) (2). However, both metrics have intrinsic limitations for early DKD detection, characterized by suboptimal sensitivity and substantial inter-individual variability, which often delay recognition of incipient renal injury (3, 4).

In recent years, a growing body of research has focused on identifying circulating and urinary biomarkers that reflect distinct pathophysiological pathways involved in DKD progression. Among the most extensively studied candidates are tumor necrosis factor receptors 1 and 2 (TNFR1/2), key mediators of inflammatory signaling (5, 6); kidney injury molecule-1 (KIM-1) and neutrophil gelatinase-associated lipocalin (NGAL), sensitive markers of tubular injury initially identified in AKI but also recognized in DKD (7–9); fibroblast growth factor 23 (FGF-23), a hormone involved in phosphate metabolism and inflammatory regulation (10); and Klotho, an anti-aging protein with pleiotropic renoprotective effects (11). Collectively, these biomarkers hold promise for adding prognostic information to risk assessment. However, despite this potential, the existing evidence regarding their ability to independently predict DKD progression remains inconclusive (10–16). More critically, most studies have included heterogeneous populations with varying degrees of renal impairment (10, 17), which may have confounded the specific prognostic value of these markers in early DKD.

This systematic review and meta-analysis was designed to evaluate the prognostic association of six circulating and urinary biomarkers (TNFR1, TNFR2, Klotho, FGF-23, KIM-1, and NGAL) with kidney function decline in patients with type 2 diabetes and relatively preserved kidney function. By restricting to prospective cohort studies with preserved kidney function at baseline, this study aimed to assess the associations between multiple biomarkers and kidney function decline, thereby informing future research directions and offering insights for risk assessment in this population.

## 2 Methods

The present systematic review and meta-analysis was performed following the Preferred Reporting Items for Systematic Reviews and Meta-Analyses (PRISMA) guidelines (18), with its protocol formally registered in the International Prospective Register of Systematic Reviews (PROSPERO) under registration number CRD420261401816.

### 2.1 Search strategy

Systematic searches were performed in PubMed, EMBASE, Web of Science, and the Cochrane Library from inception to December 31, 2025, without any language or publication date restrictions. The search strategy combined Medical Subject Headings (MeSH) terms and text words related to type 2 diabetes (e.g., “Diabetes Mellitus, Type 2”, “Diabetes Mellitus, Stable”, “Diabetes Mellitus, Noninsulin Dependent”), diabetic kidney disease (e.g., “Diabetic Nephropathy”, “Diabetic Glomerulosclerosis”, “Kimmelstiel-Wilson Disease”), biomarkers (e.g., “TNFR1”, “TNFR2”, “Klotho”, “FGF-23”, “KIM-1”, “NGAL”), and study design (e.g., “prospective cohort”, “longitudinal”, “follow-up studies”). The complete search strategy is detailed in **Supplementary Table S1**. Moreover, the reference lists of the included studies and pertinent review articles were manually examined to identify any additional eligible publications.

### 2.2 Inclusion and exclusion criteria

Studies were included if they met the following criteria: (1) adults (≥ 18 years) with type 2 diabetes; (2) relatively preserved kidney function, defined by the study’s own entry criteria as eGFR (or measured GFR) > 45 mL/min/1.73 m², or a stricter numerical threshold (e.g., >50, ≥60), or, for studies that only described the population as having preserved kidney function or normal kidney function/GFR without specifying a numerical lower threshold, a reported baseline mean or median eGFR/mGFR > 90 mL/min/1.73 m² and no predominant enrollment of patients with advanced CKD; (3) baseline measurement of at least one of the biomarkers of interest (TNFR1, TNFR2, KIM-1, FGF-23, Klotho, or NGAL) in serum, plasma, or urine (biomarkers measured in serum or plasma are hereinafter referred to as “circulating”); (4) kidney function decline clearly defined and reported as an outcome (including eGFR decline or ESRD); (5) prospective cohort design; and (6) reported HRs or ORs with 95% CIs for DKD-related outcomes, or could be calculated from the available data.

Studies were excluded if they: (a) enrolled patients with type 1 diabetes; (b) enrolled patients with non-diabetic kidney disease; (c) primarily enrolled patients with acute kidney injury; (d) employed a cross-sectional or case-control design; (e) reported only correlation coefficients (r) or linear regression coefficients (β) without effect estimates; or (f) had a sample size of < 100 participants.

### 2.3 Study selection and data extraction

The processes of study selection and data extraction were independently carried out by two reviewers, with any discrepancies addressed through consultation with a third reviewer. Data from each eligible publication were collected using a uniform extraction template, encompassing study characteristics (first author, year of publication, country, design, sample size, and follow-up period), participant demographics (mean age, gender proportion, baseline eGFR, and UACR), biomarker details (name and sample type), outcome definitions, effect estimates (HRs or ORs with corresponding 95% CIs), and covariates adjusted for in multivariate analyses.

### 2.4 Quality assessment and certainty of evidence

The methodological quality of the included studies was independently appraised by two reviewers using the Quality In Prognosis Studies (QUIPS) tool (19). Discrepancies were resolved by consultation with a third independent investigator. QUIPS assesses six domains: study participation, study attrition, prognostic factor measurement, outcome measurement, adjustment for other prognostic factors, and statistical analysis and reporting. Each domain was rated as low, moderate, or high risk of bias. Overall risk of bias was determined using the worst-rated domain approach: low overall if all domains were low; moderate overall if at least one domain was moderate and no domain was high; and high overall if any domain was high.

Overall certainty of evidence for each biomarker was appraised qualitatively using GRADE for prognosis (20–22). The initial certainty was set at moderate because all included studies were Phase 1 exploratory prognostic-factor studies. Certainty was downgraded for risk of bias, inconsistency, indirectness, imprecision, and publication bias.

### 2.5 Statistical analysis

Pooled effect estimates [hazard ratios (HRs) or odds ratios (ORs)] with corresponding 95% confidence intervals (CIs) were calculated using random-effects models. For biomarkers with studies reporting different effect measures (HR vs. OR), adjusted HRs were used for the primary analysis, and OR-reporting studies were pooled separately. The I² statistic was employed to measure inter-study variability, whereby thresholds of > 50% were interpreted as reflecting significant heterogeneity (23). Meta-analysis was performed when studies were homogeneous in effect measure (HR or OR) and outcome definition (ESRD or eGFR decline). When these criteria were met, reporting scales were then assessed: when scales were consistent, a unit-specific pooled estimate was reported; when scales differed but the direction of effect was consistent, an exploratory unitless directional summary was reported and explicitly labelled. When effect measure or outcome definition differed, results were presented descriptively. Findings for biomarkers with limited studies were interpreted as exploratory. Sensitivity analyses were considered to assess the robustness of the pooled estimates, including exclusion of studies with baseline UACR >300 mg/g, restriction to studies that adjusted for both eGFR (or measured GFR) and UACR, and exclusion of mGFR-based cohorts. These analyses were performed when the number of studies was sufficient for pooling. Because most biomarkers were informed by only one or two studies, several sensitivity analyses were not feasible; when pooling was not possible after these restrictions, results were presented descriptively. Evaluation of publication bias was not undertaken owing to the insufficient number of eligible studies for each biomarker, as the Cochrane Handbook recommends a minimum of 10 studies for reliable interpretation of funnel plots and Egger’s regression test (23). All statistical computations were performed with R software (version 4.4.2), utilizing the meta and metafor packages for data analysis.

## 3 Results

### 3.1 Study selection

The study selection procedure is illustrated in **Figure 1**. A total of 234 citations were initially retrieved through electronic database searching. Following the elimination of duplicates, 194 records were retained for further evaluation. Among these, 109 were discarded during the title and abstract review phase due to failure to satisfy the predefined inclusion criteria. The remaining 85 full-text publications were subsequently examined for eligibility. Moreover, two additional studies were uncovered through manual inspection of references lists. Ultimately, 9 investigations fulfilled all inclusion requirements and were incorporated into the present systematic review and meta-analysis.

**Figure 1:**
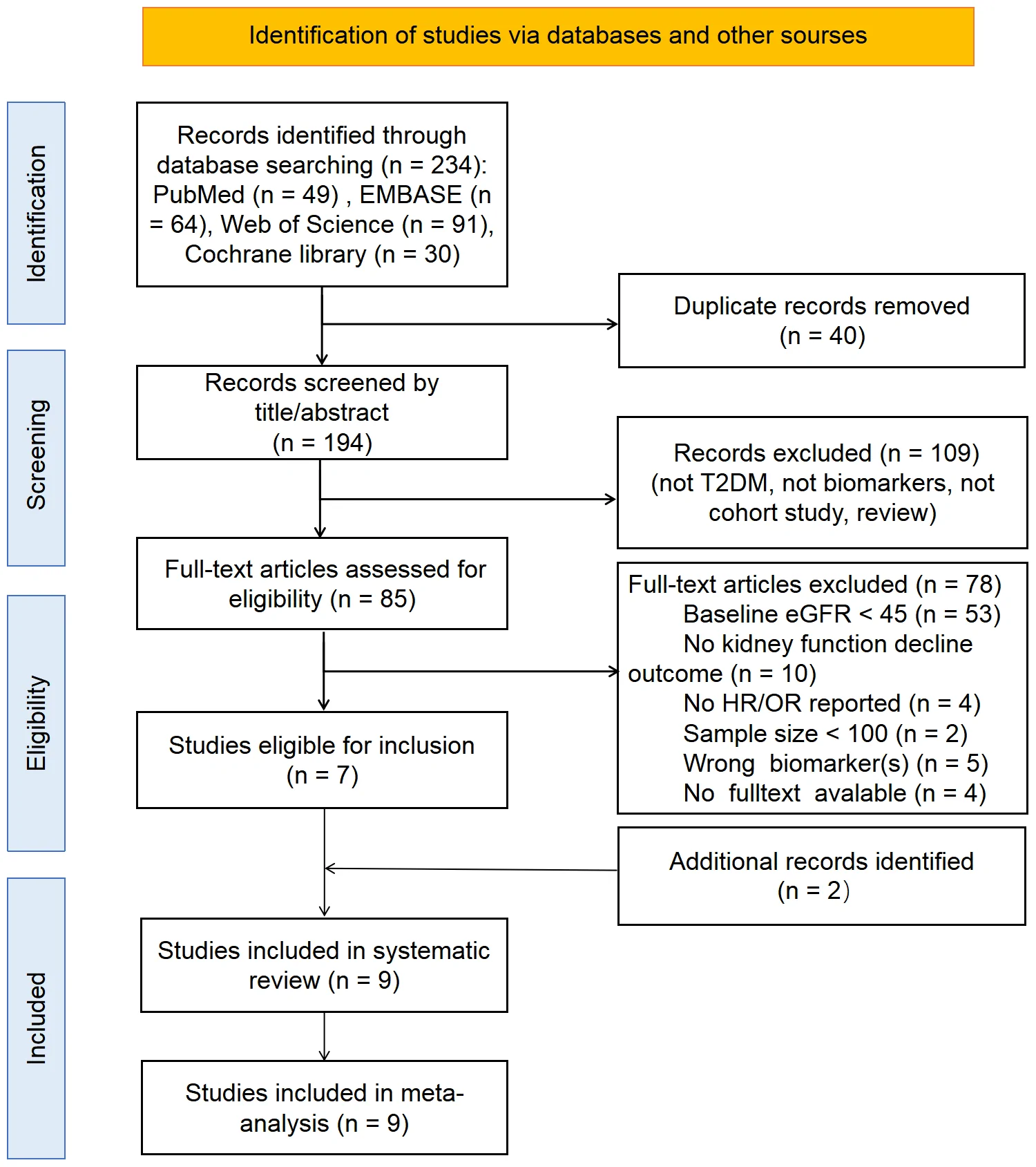
Flow diagram of study selection process. T2DM, type 2 diabetes mellitus; HR, hazard ratio; OR, odds ratio; eGFR, estimated glomerular filtration rate.

### 3.2 Study characteristics

The baseline characteristics of the nine included studies (12–15, 24–28) are summarized in **Table 1**. These investigations were published over the period from 2015 to 2025 and collectively encompassed a cohort of 4,569 participants, with individual study sample sizes ranging from 101 to 1,248. The average age of participants varied between 42.5 and 60.0 years, while the percentage of male subjects spanned from 29% to 76%. Median follow-up duration varied from 6.1 to 14.0 years. All included studies enrolled patients with relatively preserved renal function at baseline, with a minimum reported group-level mean/median baseline eGFR of 88.0 mL/min/1.73 m² (Curovic 2018, low KIM-1 group, mean ± SD: 88.0 ± 18.1) (15). The measured biomarkers investigated included circulating TNFR1, TNFR2, KIM-1, FGF-23, and Klotho, as well as urinary KIM-1 and NGAL.

**Table 1:** Study characteristics of included studies.

Study	Country/ ethnicity	Design	N	Age (years)	Male (%)	Baseline eGFR (mL/min/1.73m²)	Baseline UACR (mg/g)	Follow-up (years)	Biomarker	Sample type
Fufaa 2015 (14)	USA/American Indian	PC	260	Mean 42.5 ± 10.5	32	Mean 149 ± 45 ^a^	Median 3.2 (0.9-19.5) ^b^	Median 14.0	KIM-1, NGAL	Urine
Pavkov 2015 (24)	USA/American Indian	PC	193	Median 46 (39-53)	29	Median 120 (88- 149) ^a^	Median 72 (19-493)	Median 9.5	TNFR1, TNFR2	Serum
Curovic 2018 (15)	Denmark/ Caucasian	PC	192	Mean 58.2 ± 8.5 (high KIM-1) vs. 59.2 ± 9.1 (low KIM-1)	76	Mean 90.5 ± 17.2 high KIM-1) vs. 88.0 ± 18.1 (low KIM-1)	Median 71.0 (31.0–147.0) (high KIM-1) vs. 149.6 (63.5–510.5) (low KIM-1) ^c^	Median 6.1	KIM-1, NGAL	Urine
Fountoulakis 2018 (26)	UK/Caucasian	PC	101	Mean 60 (40-82)	59	Mean 90.7 ± 20.0	Median 24.5 (9.0-90.2) ^c^	Median 9.0	Klotho	Plasma
Frimodt-Møller 2018 (27)	Denmark/ Caucasian	PC	200	Mean 59 ± 9	76	Mean 91.1 ± 18.3	Median 103 (39–230)	Median 6.1	FGF-23	Plasma
Nowak 2018 (13)	USA/Caucasian	PC	1,032	Median 57 (51-63) (Decliners) vs. 57 (50-61) (Non-decliners)	59	Median 89 (77-99) (Decliners) vs. 97 (85-106) (Non-decliners)	Median 54 (11.7-719) (Decliners) vs. 7.2 (2.9-25) (Non-decliners)	Median 7.4	TNFR1, KIM-1, FGF-23	Plasma, urine
Niewczas 2019 PIMA cohort (25)	USA/American Indian	PC	162	Mean 45 (35-55)	72	Mean 155 ± 53 ^a^	Mean 709 ± 1772	Median 10.0	TNFR1, TNFR2	Serum
Ihara 2020 (12)	USA/Caucasian	PC	1,181	Median 57 (50-62)	58	Median 96 (83-105)	Median 13 (6-42)	Mean 7.0	TNFR1, KIM-1	Plasma
Gurung 2025 (28)	Singapore/ Asian	PC	1,248	Median 57 (50-63) (Slow) vs. 54 (49-59) (Progressive) vs. 58 (50-63) (Accelerated)	49	Median 99-102	Median 13 (4-36) (Slow) vs. 27 (6-66) (Progressive) vs. 51 (19-141) (Accelerated)	Median 9.4	KIM-1	Plasma

Values are presented as mean (range), mean ± SD, median (IQR), or median (range) as reported in each study.

Studies marked with ^a^ used iothalamate glomerular filtration rate (mGFR) for GFR measurement; all other studies used eGFR.

Studies marked with ^b^ reported UACR in mg/mmol (1 mg/mmol ≈ 8.84 mg/g).

Studies marked with ^c^ reported UAER (μg/min) rather than UACR.

N, number of participants; PC, prospective cohort; eGFR, estimated glomerular filtration rate; UACR, urine albumin-to-creatinine ratio; NOS, Newcastle-Ottawa Scale.

**Table 2** summarizes the outcome measures, event numbers, extracted effect estimates, and main covariates adjusted for in multivariate analyses for each included study. Outcome measures comprised eGFR decline and ESRD.

**Table 2:** Effect estimates extracted from included studies.

Study	Biomarker	Sample	Outcome (definition)	Events (n)	Original estimate (95% CI)	P value	Method of calculation	Variables adjusted
Fufaa 2015 (14)	KIM-1, NGAL	Urine	ESRD: initiation of renal replacement therapy or death from diabetic kidney disease	74	KIM-1: 0.95 (0.71-1.28)	>0.05	Per 1-SD increase	age, sex, diabetes duration, hypertension, HbA_1c_, GFR, ACR
					NGAL: 1.59 (1.20-2.11)	0.001		
Pavkov 2015 (24)	TNFR1, TNFR2	Serum	ESRD: dialysis, kidney transplant, or death attributed to diabetic kidney disease	62	TNFR1: 1.60 (1.10-2.20)	<0.05	Per IQR increase	age, sex, MAP, HbA_1c_, ACR, mGFR
					TNFR2: 1.7 (1.2-2.3)	<0.05		
Curovic 2018 (15)	KIM-1, NGAL	Urine	eGFR decline >30% from baseline	39	KIM-1: 1.68 (1.04-2.71)	0.034	Per 1-SD increase	sex, age, LDL cholesterol, smoking, HbA_1c_, plasma creatinine, SBP, urinary AER
					NGAL: 1.29 (0.89–1.86)	0.17		
Fountoulakis 2018 (26)	Klotho	Plasma	eGFR decline >50% from baseline	22	0.28 (0.15-0.52)	<0.001	Per 10% increase	age, sex, baseline eGFR, SBP, AER
Frimodt-Møller 2018 (27)	FGF-23	Plasma	eGFR decline >30% from baseline ^a^	42	0.98 (0.68-1.41)	0.90	Per 1-SD increase	sex, age, LDL cholesterol, smoking, HbA_1c_, plasma creatinine, SBP, urinary AER, 25(OH)-D vitamin
Nowak 2018 (13)	TNFR1, KIM-1, FGF-23	Plasma, urine	eGFR decline ≥30% from baseline within 5 years	114	TNFR1 (Plasma): 1.42 (1.10-1.84)	<0.01	Per quartile increase	HbA_1c_, BMI, SBP, eGFR, ACR
					KIM-1 (Plasma): 1.41 (1.13-1.75)	<0.01		
					FGF-23 (Plasma): 0.96 (0.78-1.18)	>0.05		
					KIM-1 (Urine): 0.94 (0.74-1.19)	>0.05		
Niewczas 2019 PIMA cohort (25)	TNFR1, TNFR2	Serum	ESRD: need for dialysis, kidney transplantation, or death from diabetic kidney disease	38	TNFR1: 1.71 (1.07-2.72)	<0.01	Per tertile increase	age, GFR, HbA_1c_, ACR, gender, diabetes duration, SBP, BMI
					TNFR2: 2.32 (1.33-4.06)	<0.01		
Ihara 2020 (12)	TNFR1, KIM-1	Plasma	Rapid eGFR decline: annual eGFR loss ≥5 mL/min/1.73m²	152	TNFR1: 1.16 (0.93-1.46)	>0.05	Per quartile increase	eGFR, SBP, ACR, HbA_1c_, fibrosis index
					KIM-1: 1.36 (1.13-1.65)	<0.05		
Gurung 2025 (28)	KIM-1	Plasma	Accelerated eGFR decline: fast progressive trajectory by LCMM	51	2.95 (2.01-4.32)	<0.001	Per 1-SD increase	age, sex, ethnicity, baseline eGFR, ACR, HbA_1c_, diabetes duration, BMI, SBP, triglycerides

ACR, albumin-to-creatinine ratio; AER, albumin excretion rate; BMI, body mass index; CI, confidence interval; eGFR, estimated glomerular filtration rate; IQR, interquartile range; LCMM, latent class mixed model; LDL, low-density lipoprotein; MAP, mean arterial pressure; mGFR, measured glomerular filtration rate; OR, odds ratio; SBP, systolic blood pressure; SD, standard deviation.

Studies marked with ^a^ used eGFR decline >30% as one of three predefined endpoints, with mortality as the primary framing.

### 3.3 Quality assessment

Methodological quality was assessed using the QUIPS tool, with domain-level ratings for each biomarker presented in **Table 3**. Overall, most studies had a moderate risk of bias. Study participation, outcome measurement, and adjustment for other prognostic factors were rated low risk in all studies, as they clearly defined the source population, used validated outcome definitions, and adjusted for key confounders including age, sex, and measures of kidney function and albuminuria (either eGFR and UACR, or plasma creatinine and urinary AER). Study attrition was rated moderate for most studies because they did not report attrition rates. Only Frimodt-Møller 2018 (27) and Curovic 2018 (15) explicitly reported 0% loss to follow-up and were rated low. Prognostic factor measurement was rated moderate for most studies due to incomplete reporting of assay platforms, storage duration, or freeze-thaw cycles, whereas Fufaa 2015 (14) was rated high risk for urinary KIM-1 and NGAL because urine samples were stored for 12–21 years before measurement. Statistical analysis and reporting was rated moderate for all studies except Fountoulakis 2018 (26), which was rated low because it explicitly used competing risk models. The other studies did not address competing risk of death. Consequently, the overall risk of bias was moderate for most studies, and high for Fufaa 2015 (14) in the urinary KIM-1 and NGAL analyses.

**Table 3:** QUIPS risk of bias assessment by biomarker of included studies.

Biomarker	Study	Study participation	Study attrition	Prognostic factor measurement	Outcome measurement	Adjustment for other prognostic factors	Statistical analysis and reporting	Overall RoB
Circulating TNFR1	Pavkov 2015 (24)	Low	Moderate	Moderate	Low	Low	Moderate	Moderate
	Nowak 2018 (13)	Low	Moderate	Moderate	Low	Low	Moderate	Moderate
	Niewczas 2019 PIMA cohort (25)	Low	Moderate	Moderate	Low	Low	Moderate	Moderate
	Ihara 2020 (12)	Low	Moderate	Moderate	Low	Low	Moderate	Moderate
Circulating TNFR2	Pavkov 2015 (24)	Low	Moderate	Moderate	Low	Low	Moderate	Moderate
	Niewczas 2019 PIMA cohort (25)	Low	Moderate	Moderate	Low	Low	Moderate	Moderate
Circulating Klotho	Fountoulakis 2018 (26)	Low	Moderate	Low	Low	Low	Low	Moderate
Circulating FGF-23	Frimodt-Møller 2018 (27)	Low	Low	Low	Low	Low	Moderate	Moderate
	Nowak 2018 (13)	Low	Moderate	Moderate	Low	Low	Moderate	Moderate
Circulating KIM-1	Nowak 2018 (13)	Low	Moderate	Moderate	Low	Low	Moderate	Moderate
	Ihara 2020 (12)	Low	Moderate	Moderate	Low	Low	Moderate	Moderate
	Gurung 2025 (28)	Low	Moderate	Low	Low	Low	Moderate	Moderate
Urinary KIM-1	Fufaa 2015 (14)	Low	Moderate	High	Low	Low	Moderate	High
	Nowak 2018 (13)	Low	Moderate	Moderate	Low	Low	Moderate	Moderate
	Curovic 2018 (15)	Low	Low	Moderate	Low	Low	Moderate	Moderate
Urinary NGAL	Fufaa 2015 (14)	Low	Moderate	High	Low	Low	Moderate	High
	Curovic 2018 (15)	Low	Low	Moderate	Low	Low	Moderate	Moderate

### 3.4 Results of biomarker analyses

Findings for each biomarker are summarized in **Table 4**. Circulating TNFR1, TNFR2, and KIM-1 showed potential positive associations with kidney function decline, whereas higher circulating Klotho levels were associated with a lower risk. No significant associations were found for circulating FGF-23. Findings for urinary NGAL and urinary KIM-1 were inconsistent across studies. Because most biomarkers were supported by only two or three studies, and several could not be pooled because of differences in effect measures, exposure scales, and outcome definitions, results are presented descriptively in the following sections. Even when pooling was performed, each pooled analysis was based on only two studies, so the effect estimates, heterogeneity assessments, and prediction intervals may be unstable and should be interpreted as exploratory.

**Table 4:** Summary of included biomarkers and pooling strategy.

Biomarker	No. of studies	Effect measure	Exposure scale	Pooled?	Pooled estimate (if applicable)	95% CI	I²	Remarks
Circulating TNFR1 (HR group)	2 (24, 25)	HR	per IQR vs. per tertile	Directional summary	HR = 1.64	1.24 to 2.16	0%	Unitless, directional only
Circulating TNFR1 (OR group)	2 (12, 13)	OR	per quartile	Yes	OR = 1.27	1.04 to 1.55	25.5%	Exploratory
Circulating TNFR2	2 (24, 25)	HR	per IQR vs. per tertile	Directional summary	HR = 1.82	1.40 to 2.38	0%	Unitless, directional only
Circulating Klotho	1 (26)	HR	per 10% increase	No	—	—	—	Single study, descriptive
Circulating FGF-23	2 (13, 27)	HR + OR	per 1-SD vs. per quartile	No	—	—	—	Different scales/effect measures, descriptive
Circulating KIM-1	2 (12, 13) + 1 (28)	OR	per quartile (2) + categorical trajectory (1)	Partially pooled	OR = 1.38	1.20 to 1.59	0%	Only 2 per-quartile studies pooled; Gurung described separately (different outcome definition)
Urinary KIM-1	3 (13–15)	HR + OR	per 1-SD vs. per quartile	No	—	—	—	Different scales/directions/outcomes, descriptive
Urinary NGAL	2 (14, 15)	HR	per 1-SD	No	—	—	—	Different outcome definitions, descriptive

#### 3.4.1 Circulating biomarkers

##### 3.4.1.1 Circulating TNFR1

A total of four studies (12, 13, 24, 25) involving 2,568 patients contributed to this analysis. Despite differences in effect measures (HR vs. OR), reporting scales (per IQR, per quartile, per tertile), and outcome definitions (ESRD vs. eGFR decline), all four studies showed positive associations between circulating TNFR1 levels and the risk of kidney function decline, with three of them (13, 24, 25) reaching statistical significance. Two HR-reporting studies [Pavkov 2015 (24): HR = 1.60, 95% CI: 1.10 to 2.20; Niewczas 2019 (25): HR = 1.71, 95% CI: 1.07 to 2.72], both using ESRD as the outcome and per IQR and per tertile increases respectively, yielded a pooled HR of 1.64 (95% CI: 1.24 to 2.16; prediction interval: 0.27 to 9.95; I² = 0%; **Figure 2**). Given the small number of studies and different exposure scales, this estimate is presented as a directional summary rather than a unit-specific effect. Sensitivity analyses excluding the study (25) with UACR > 300 mg/g left only Pavkov 2015 (24), which still showed a positive association (HR = 1.60, 95% CI: 1.10 to 2.20). In addition, both studies used mGFR for GFR measurement; therefore, excluding mGFR-based cohorts was not feasible. Two OR-reporting studies [Nowak 2018 (13): OR = 1.42, 95% CI: 1.10 to 1.84; Ihara 2020 (12): OR = 1.16, 95% CI: 0.93 to 1.46), both using eGFR decline as the outcome and per quartile increase, yielded a pooled OR of 1.27 (95% CI: 1.04 to 1.55; prediction interval: 0.26 to 6.13; I² = 25.5%; **Figure 3**). None of the sensitivity analyses changed the pooled OR, as both studies met the criteria. All results are based on two-study meta-analyses and should be interpreted as exploratory.

**Figure 2:**
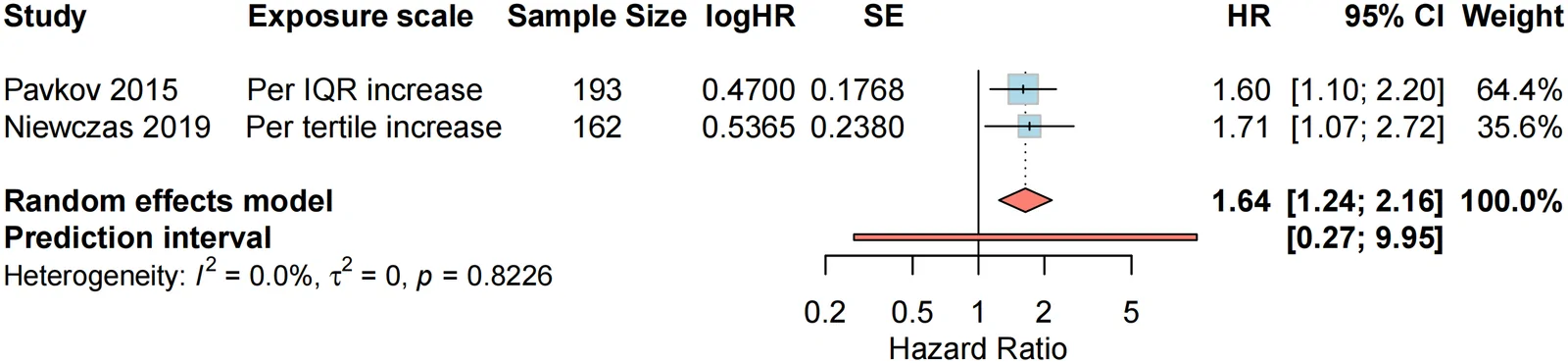
Forest plot of circulating TNFR1 (HR-reporting studies) levels and the risk of kidney function decline. Two HR-reporting studies were pooled as an exploratory unitless directional summary (HR = 1.64, 95% CI 1.24 to 2.16; prediction interval 0.27 to 9.95; I² = 0%). Random-effects model. Because only two studies were included and exposure scales differed (per IQR vs. per tertile), this estimate should be interpreted as exploratory.

**Figure 3:**
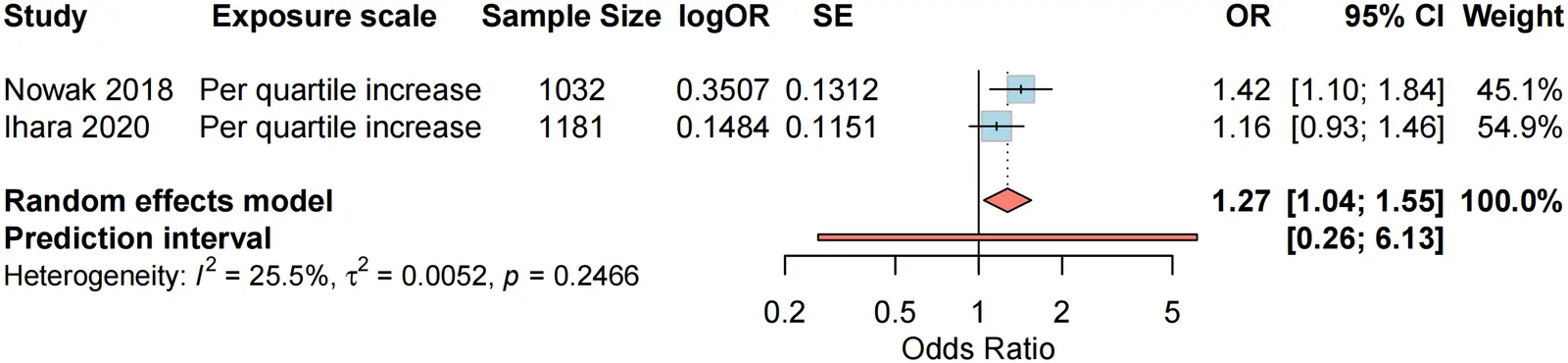
Forest plot of circulating TNFR1 (OR-reporting studies) levels and the risk of kidney function decline. Two OR-reporting studies were pooled on a per-quartile scale (OR = 1.27, 95% CI 1.04 to 1.55; prediction interval 0.26 to 6.13; I² = 25.5%). Random-effects model. This estimate is exploratory given the small number of studies.

##### 3.4.1.2 Circulating TNFR2

Two studies (24, 25) with a total of 355 patients were included. Both studies reported HRs for ESRD, with Pavkov 2015 (24) reporting per IQR increase (HR = 1.70, 95% CI: 1.20 to 2.30) and Niewczas 2019 (25) reporting per tertile increase (HR = 2.32, 95% CI: 1.33 to 4.06). An exploratory meta-analysis yielded a pooled HR of 1.82 (95% CI: 1.40 to 2.38; prediction interval: 0.33 to 10.17; I² = 0%; **Figure 4**), which should be interpreted as a directional summary due to the small number of studies and different reporting scales. In addition, to evaluate the potential influence of baseline albuminuria, we excluded the study with UACR > 300 mg/g (25); the remaining single study (24) still demonstrated a positive association (HR = 1.70, 95% CI: 1.20 to 2.30). Both studies were mGFR cohorts; therefore, exclusion of mGFR cohorts was not feasible.

**Figure 4:**
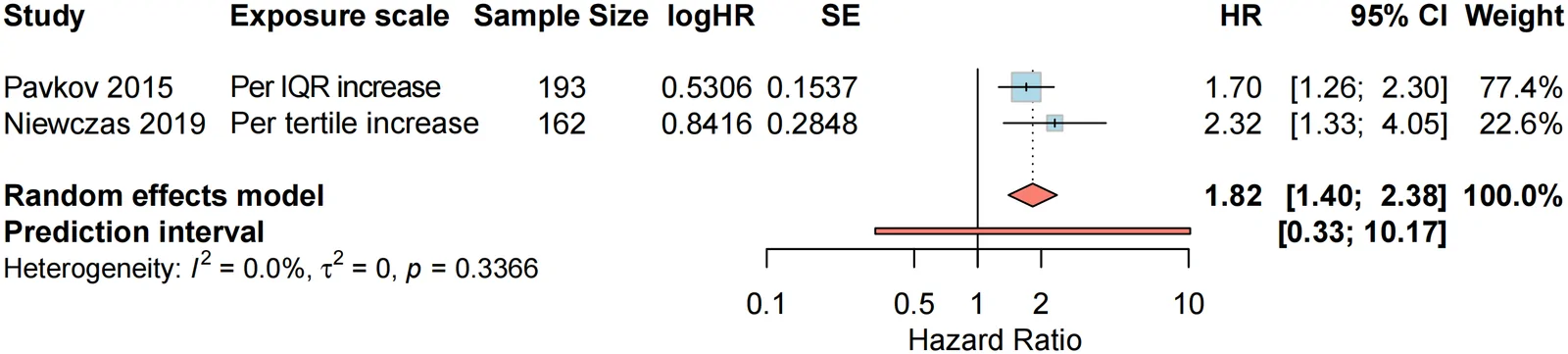
Forest plot of circulating TNFR2 levels and the risk of kidney function decline. Two HR-reporting studies were pooled as an exploratory unitless directional summary (HR = 1.82, 95% CI 1.40 to 2.38; prediction interval 0.33 to 10.17; I² = 0%). Random-effects model. Because only two studies were included and exposure scales differed (per IQR vs. per tertile), this estimate should be interpreted as exploratory.

##### 3.4.1.3 Circulating Klotho

One study (26), with 101 participants, met the inclusion criteria. In this study, higher circulating Klotho levels were significantly associated with a reduced risk of eGFR decline > 50% (HR = 0.28, 95% CI: 0.15 to 0.52, p < 0.01). Although the single included study adjusted for eGFR and AER (rather than UACR), sensitivity analyses were not feasible. As this finding is based on a single study, it is presented descriptively and should be interpreted with caution.

##### 3.4.1.4 Circulating FGF-23

Two studies (13, 27) comprising 1,232 participants, were included. Both studies evaluated the association between circulating FGF-23 levels and kidney function decline. Frimodt-Møller 2018 (27) reported a per 1-SD increase (HR = 0.98, 95% CI: 0.68 to 1.41), adjusting for plasma creatinine and urinary AER, for eGFR decline >30%, and Nowak 2018 (13) reported a per quartile increase (OR = 0.96, 95% CI: 0.78 to 1.18), adjusting for eGFR and UACR, for eGFR decline ≥30% within 5 years. Both studies showed null associations, with effect estimates close to unity and confidence intervals crossing 1.0. Given the differences in effect measures, reporting scales, and adjustment sets, as well as the limited number of studies, meta-analysis was not performed. Consequently, sensitivity analysis restricting to studies adjusted for eGFR and UACR was not feasible. These findings are presented descriptively and should be interpreted as exploratory.

##### 3.4.1.5 Circulating KIM-1

Three studies (12, 13, 28) comprising 3,461 participants reported positive and statistically significant associations between circulating KIM-1 levels and kidney function decline (all p < 0.05). Two studies using per quartile ORs [Nowak 2018 (13): OR = 1.41, 95% CI: 1.13 to 1.75; Ihara 2020 (12): OR = 1.36, 95% CI: 1.13 to 1.65), both with eGFR decline outcomes, yielded a pooled OR of 1.38 (95% CI: 1.20 to 1.59; prediction interval: 0.55 to 3.49; I² = 0%; **Figure 5**). Sensitivity analyses yielded consistent results, as both studies met the criteria. A third study (Gurung 2025) (28), which used a latent class mixed model to define accelerated versus slow eGFR decline trajectory rather than conventional eGFR decline thresholds, reported a substantially higher and statistically significant OR of 2.95 (95% CI: 2.01 to 4.32). Due to differences in outcome definition (LCMM trajectory vs. conventional eGFR decline thresholds) and exposure scale (categorical trajectory classification vs. per quartile), this study was not pooled with the other two. The pooled OR from the two per-quartile studies should be interpreted as exploratory, as it is based on a limited number of studies.

**Figure 5:**
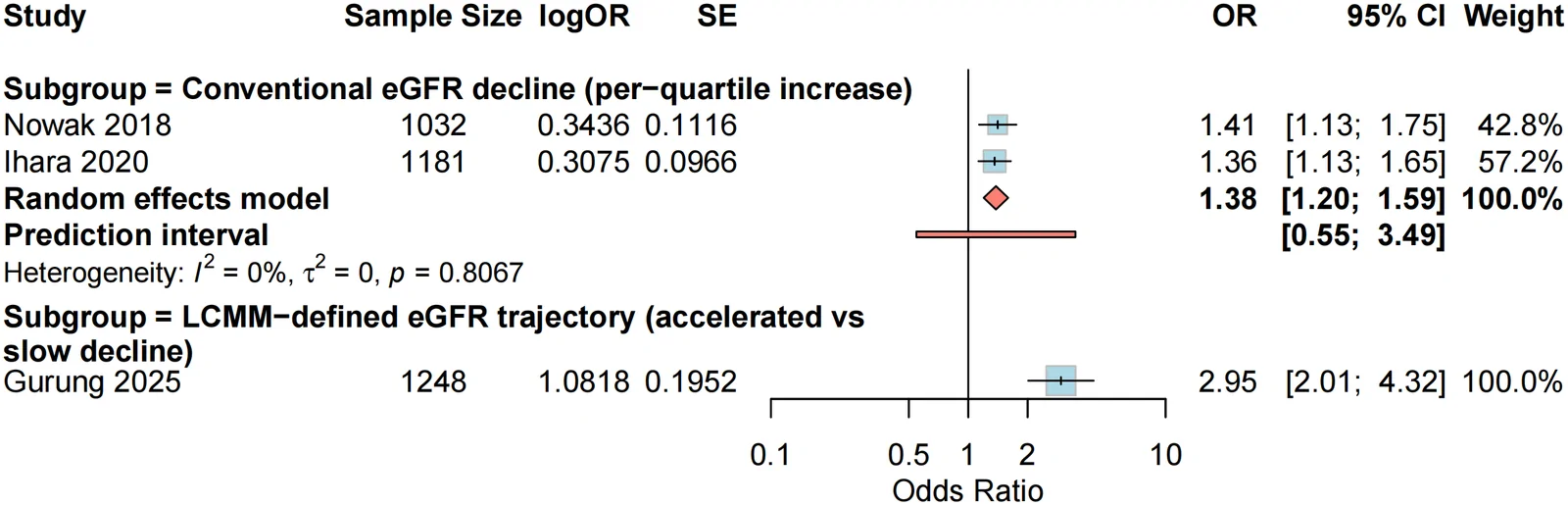
Forest plot of circulating KIM-1 and the risk of kidney function decline. Two per-quartile OR studies were pooled (OR = 1.38, 95% CI 1.20 to 1.59; prediction interval 0.55 to 3.49; I² = 0%). A third study (Gurung 2025), which used a latent class mixed model, was not pooled (OR = 2.95, 95% CI 2.01 to 4.32). Random-effects model for the pooled estimate. The pooled estimate is exploratory.

#### 3.4.2 Urinary biomarkers

##### 3.4.2.1 Urinary KIM-1

For urinary KIM-1, three studies (13–15) involving 1,484 participants reported associations with kidney function decline, but the findings were inconsistent across studies, with two showing no significant association and one reporting a significant positive association. Among the two studies using per 1-SD HRs, Fufaa 2015 (14) showed no significant association with ESRD (HR = 0.95, 95% CI: 0.71 to 1.28), whereas Curovic 2018 (15) reported a significant positive association with eGFR decline >30% (HR = 1.68, 95% CI: 1.04 to 2.71). The third study, Nowak 2018 (13), reported a non-significant per-quartile OR of 0.94 (95% CI: 0.74 to 1.19) for eGFR decline ≥30%. These three studies differed in outcome definition, effect measure, and exposure scale, and the direction of effect was also inconsistent. Given such heterogeneity across multiple domains, these estimates were not pooled. Therefore, sensitivity analyses were not feasible because no pooling was possible even after excluding Curovic 2018 (adjusted for plasma creatinine and urinary AER) (15) or Fufaa 2015 (used mGFR) (14). Overall, the current evidence does not support urinary KIM-1 as a consistent prognostic marker for kidney function decline in patients with preserved kidney function.

##### 3.4.2.2 Urinary NGAL

Two studies (14, 15) involving 452 patients examined the association between urinary NGAL levels and the risk of kidney function decline. Fufaa 2015 (14) reported a statistically significant positive association for ESRD (HR = 1.59, 95% CI: 1.20 to 2.11); Curovic 2018 (15) reported a positive but non-significant association for eGFR decline >30% (HR = 1.29, 95% CI: 0.89 to 1.86). Both studies used HRs on a per 1-SD scale but were not pooled owing to different outcome definitions. Sensitivity analyses left only one study each [Fufaa 2015 (14) for adjusted restriction; Curovic 2018 (15) for mGFR exclusion], precluding pooling. Overall, both studies reported positive point estimates, but only one reached statistical significance. The evidence for urinary NGAL remains limited and inconclusive.

### 3.5 Certainty of evidence

Certainty of evidence for each biomarker was assessed using GRADE for prognosis (**Supplementary Table S2**). Initial certainty was set at moderate because all included studies were Phase 1 exploratory prognostic-factor studies (20–22). All biomarkers were ultimately rated very low certainty. The main reasons were risk of bias (most studies did not address competing risk of death), indirectness (mixed effect measures, exposure scales, and outcome definitions), and imprecision (few studies per biomarker, ranging from 1 to 4). Publication bias could not be assessed because fewer than 10 studies were available for each biomarker.

## 4 Discussion

### 4.1 Main findings

The clinical utility of biomarkers in risk assessment for kidney function decline in type 2 diabetes has garnered growing interest. This systematic review and meta-analysis evaluated six circulating and urinary biomarkers for their association with kidney function decline among individuals with T2DM and relatively preserved kidney function. Circulating TNFR1, TNFR2, and KIM-1, showed potential positive associations, whereas Klotho was inversely associated. No significant associations were observed for circulating FGF-23. Findings for urinary NGAL and urinary KIM-1 were inconsistent across studies. However, all findings were rated very low certainty and should be considered exploratory.

Another notable finding was the limited evidence base. The target population was defined as T2DM with relatively preserved kidney function (eGFR or measured GFR >45 mL/min/1.73 m²), reflecting our focus on the window for early intervention before irreversible kidney function decline. Only a limited number of studies met these broader criteria, and when stricter early DKD criteria (eGFR ≥60 mL/min/1.73 m² with UACR <300 mg/g) were applied, even fewer studies were eligible (12, 14, 26–28). This suggests an evidence gap in early DKD research. In addition, to address heterogeneity related to macroalbuminuria, we explored sensitivity analyses excluding studies with UACR >300 mg/g, but these were also limited because only one study remained, precluding formal sensitivity analysis. These evidence gaps highlight the need for larger, standardized prospective studies in strictly defined early DKD.

### 4.2 Potential role of biomarkers

#### 4.2.1 Inflammatory mediators TNFR1 and TNFR2

Circulating TNFR1 and TNFR2 showed consistent positive directions of effect across included studies, although the evidence was rated very low certainty and should be considered exploratory. For TNFR1, both the HR-reporting and OR-reporting studies yielded positive pooled estimates, although the two groups were analyzed separately because of different effect measures and outcome definitions. For TNFR2, the two HR-reporting studies also yielded a positive pooled estimate. This is broadly consistent with the meta-analysis by Cheng et al. (2020) (6). TNFR1 and TNFR2 are the primary receptors for tumor necrosis factor-α (TNF-α) and play critical roles in inflammatory signal transduction. In the kidney, TNFR1 is predominantly expressed in glomerular and tubular endothelial cells, whereas TNFR2 expression is largely confined to immune cells and endothelial cells (5). Experimental studies have shown that and TNF-α expression increases in glomerular and tubular cells following diabetes induction (29), TNF-α mediates kidney injury through binding to its soluble receptors, sTNFR1 and sTNFR2. Administration of a TNFR: Fc fusion protein in diabetic rat models reduced urinary TNF-α excretion and mitigated renal hypertrophy (30). Consistently, clinical studies have shown that elevated TNFR levels are associated with rapid kidney function decline years before the onset of overt nephropathy (31). These findings support the potential role of the TNF-TNFR pathway in DKD progression.

#### 4.2.2 Mineral metabolism and anti-aging regulators FGF-23 and Klotho

FGF-23 and Klotho are central regulators of mineral metabolism and anti-aging processes. FGF-23 is an osteocyte-derived endocrine factor implicated in DKD progression through multiple pathways, including regulation of calcium-phosphate metabolism (32), promotion of inflammation and fibrosis (33–35), and possibly ACE2 suppression with subsequent renin-angiotensin-aldosterone system (RAAS) activation (36–38). In the present review, two studies evaluated circulating FGF-23 and both showed null associations (13, 27). A recent meta-analysis found no statistically significant link between FGF-23 and the likelihood of developing DKD (p = 0.65) (10), whereas a broader meta-analysis (17) reported a positive association (p < 0.05). Therefore, its role in early DKD remains uncertain. Several factors may explain the negative finding in our relatively preserved kidney function population. First, FGF-23 levels typically rise substantially after renal function impairment has occurred (27), and may not be elevated enough in patients with preserved eGFR to show a clear association. Second, RAAS inhibitors, commonly used in this population, may influence FGF-23 levels and attenuate its association with DKD risk (39). Third, the limited number of studies may have affected the precision of our estimate. These findings suggest that FGF-23 has limited utility for risk assessment in this population, and larger prospective studies are needed to clarify its role.

Klotho is an anti-aging protein predominantly expressed in distal renal tubules, with pleiotropic effects including anti-oxidation, anti-fibrosis, anti-inflammation, and regulation of phosphate metabolism via its role as a co-receptor for FGF-23 (40–42). Experimental studies have demonstrated renoprotective properties of Klotho in diabetic models (43, 44). Clinically, a meta-analysis by Xin et al. (2022) (45) reported that soluble Klotho levels were significantly lower in patients with diabetic nephropathy than in those without nephropathy (SMD = −1.52, 95% CI: −2.24 to −0.80), with the difference already evident in early DKD. In the present review, one study (26) reported that higher circulating Klotho levels were associated with a reduced risk of eGFR decline (HR = 0.28, 95% CI: 0.15 to 0.52). Due to the inclusion of only one study, this finding should be interpreted as exploratory and requires validation in larger cohorts.

#### 4.2.3 Tubular injury markers KIM-1 and NGAL

KIM-1 and NGAL are established markers of tubular injury. KIM-1 is a marker of tubular injury initially identified in AKI, but has since been recognized in chronic settings such as DKD (8). For circulating KIM-1, all three studies reported significant positive associations (12, 13, 28). Two per-quartile OR studies (12, 13) were pooled (OR = 1.38, 95% CI 1.20 to 1.59), and the third also showed a significant association (28). In contrast, the three urinary KIM-1 studies showed inconsistent directions (13–15), and the available evidence does not support urinary KIM-1 as a consistent prognostic marker in early DKD. This finding is consistent with the meta-analysis by Liu et al. (17), which also reported a stronger association for plasma KIM-1 than for urinary KIM-1. The prognostic value of KIM-1 thus appears to differ by sample type, which may reflect differences in biological origins. KIM-1 is upregulated on proximal tubular epithelial cells after injury, and its extracellular domain can be cleaved and released into both urine and circulation (8). Urinary KIM-1 mainly reflects apical tubular injury. However, in early-to-mid DKD, tubular injury is chronic and interstitial. Loss of tubular cell polarity and increased transepithelial permeability cause KIM-1 to enter the interstitium and circulation rather than the tubular lumen (46). Consequently, urinary KIM-1 is less sensitive in early DKD, whereas circulating KIM-1 captures ongoing interstitial injury. In addition, circulating KIM-1 has extrarenal sources, particularly CD8^+^ T cells, integrating local damage with systemic inflammation (47). Circulating KIM-1 elevation can precede urinary KIM-1 elevation, suggesting it reflects an earlier or more systemic disease process (47). Additionally, Coca et al. (46) reported that baseline plasma KIM-1 independently predicted eGFR decline and improved prediction model performance in both early and established DKD. Collectively, these findings suggest that plasma KIM-1 may serve as a promising indicator for early identification of high-risk patients compared with urinary KIM-1. However, this interpretation remains speculative, as our meta-analysis did not directly compare both sample types within matched populations. Head-to-head studies are needed to validate these findings.

NGAL was initially studied in the context of inflammation and AKI, but its role has since been extended to chronic kidney disease, including DKD (9, 48). In DKD, urinary NGAL is primarily derived from injured proximal tubular epithelial cells and is involved in the regulation of tubular cell proliferation and apoptosis (9, 48). Notably, elevated NGAL levels have been detected even in diabetic patients with normoalbuminuria (49). In the present review, two studies showed positive directions of effect (14, 15), which is consistent with prior studies (17, 50). However, because the two studies used different outcome definitions, they were not pooled. Fufaa 2015 (14) reported a significant positive association with ESRD, whereas Curovic 2018 (15) reported a positive but non-significant association with eGFR decline > 30%. This discrepancy may partly reflect the different outcome definitions and may also be related to the susceptibility of NGAL to confounding from systemic inflammation and cardiovascular disease, which can limit its specificity for kidney injury (9). Therefore, the current evidence for urinary NGAL as a prognostic marker in this population remains limited, and larger prospective studies are needed to validate its utility.

Beyond the direction of associations, we also assessed whether these biomarkers provide incremental prognostic value beyond eGFR and UACR. Several included studies adjusted for baseline eGFR/mGFR and UACR/AER, and some biomarkers remained associated with kidney function decline after adjustment. For example, adding TNFR1 or TNFR2 improved the C-index for ESRD in Pavkov 2015 (from 0.858 to 0.873 and 0.879, respectively). In Nowak 2018, a multi-marker model including TNFR1, plasma KIM-1, and urinary EGF/MCP-1 ratio improved the AUC from 0.71 with ACR alone to 0.81. Fufaa 2015 found only small increases in c-statistic for urinary NGAL and L-FABP (0.828 to 0.833 and 0.832), with uncertain clinical significance. In contrast, Curovic 2018 found urinary KIM-1 improved mortality prediction but not eGFR decline. However, incremental value beyond eGFR and UACR was modest and inconsistently reported. Most studies did not simultaneously report NRI, IDI, and decision curve analysis, and external validation was rare. Therefore, current evidence is insufficient to recommend routine clinical use of these biomarkers for risk stratification in patients with relatively preserved kidney function. Future studies should report standardized incremental performance metrics and validate multi-biomarker models in independent cohorts.

### 4.3 Strengths and limitations

Several strengths of this study merit mention. First, unlike Liu et al. (17), which included various CKD etiologies and different stages of kidney function, and Wang et al. (10), which did not restrict kidney function stage, our inclusion criteria focused specifically on T2DM with relatively preserved kidney function (eGFR or mGFR >45 mL/min/1.73 m²), addressing a clinically relevant window before irreversible decline. Second, unlike Wang et al. (10), which included multiple observational study designs, we restricted inclusion to prospective cohort studies, thereby minimizing confounding and improving temporality at the design level. Third, unlike several previous meta-analyses that evaluated only a single biomarker, including Cheng et al. (6) for TNFR1/2, Wang et al. (10) for FGF-23, Yu et al. (11) for Klotho, and Prashant et al. (51) for NGAL, our review simultaneously evaluates six circulating and urinary biomarkers and explicitly restricts the kidney function range, providing evidence-based guidance for clinical prioritization.

Several limitations should be acknowledged. First, the number of studies was limited. For several biomarkers, only one or two studies were available (Klotho: one study; TNFR2: two studies; FGF-23: two studies; urinary NGAL: two studies). Among these, Klotho was based on a single study, and FGF-23 and urinary NGAL could not be quantitatively pooled because of differences in effect measures, exposure scales, or outcome definitions. Therefore, their findings should be considered exploratory. In addition, because fewer than 10 studies were available for each biomarker (range 1-4), publication bias could not be quantitatively assessed using funnel plots or Egger’s regression test, and therefore cannot be excluded (23). All findings were rated very low certainty by GRADE, mainly due to risk of bias, indirectness, and imprecision. Future high-quality prospective studies are needed to corroborate these findings.

Second, notable between-study differences were observed across multiple methodological domains, which may have influenced effect estimates and limited comparability. These differences directly precluded quantitative pooling of all studies for several biomarkers, including circulating TNFR1, FGF-23, circulating and urinary KIM-1, and urinary NGAL. For some biomarkers, such as TNFR2, pooling was possible only as a unitless directional summary. Specifically, outcome definitions differed (ESRD vs. different eGFR decline thresholds, including LCMM-defined trajectories), effect measures were inconsistent (HR vs. OR), and exposure scales varied (per 1-SD, per quartile, or categorical trajectory). Adjustment sets also differed: most studies adjusted for eGFR and UACR, but two used plasma creatinine and urinary AER (15, 27), and the only Klotho study adjusted for eGFR and AER (26). Most studies used eGFR, whereas three used mGFR (14, 24, 25). Assay platforms and sample storage conditions also varied: circulating KIM-1 was measured using ELISA or Olink, urinary KIM-1 and NGAL were measured on different platforms, and Fufaa 2015 (14) measured urinary samples after 12–21 years of storage. Follow-up durations ranged from 6.1 to 14.0 years. Although we restricted quantitative pooling to studies with consistent outcome definitions and effect measures and attempted to exclude studies with different adjustment sets or mGFR-based cohorts, these analyses were limited by the small number of studies. Therefore, residual confounding cannot be excluded, and these findings should be interpreted as exploratory.

Third, most included studies did not use competing risk models for death. For example, Fufaa 2015 (14) defined ESRD as initiation of renal replacement therapy or death from diabetic kidney disease, embedding death in the primary outcome. Frimodt-Møller 2018 (27) used a composite renal endpoint combining eGFR decline and all-cause mortality. Curovic 2018 (15) reported all-cause mortality as a co-primary endpoint. Only Fountoulakis 2018 (26) explicitly used competing risk models. Failure to account for competing risk may have biased the associations across all studies that did not use these models.

Finally, the observed differences between circulating and urinary KIM-1 were based on separate study populations rather than direct matched comparisons. Head-to-head studies measuring both sample types in the same individuals are needed.

Despite these limitations, the overall direction of the associations was broadly consistent with previous independent studies (6, 7, 10, 51). However, given the very low certainty of evidence and the observational nature of the data, causality cannot be inferred, and further validation in large-scale cohorts is warranted. Future studies should also develop and validate multi-biomarker prediction models with appropriate validation frameworks.

## 5 Conclusion

In summary, this review evaluated six circulating and urinary biomarkers for kidney function decline in T2DM with relatively preserved kidney function. Circulating TNFR1, TNFR2, and KIM-1 showed potential positive associations, whereas one study reported an inverse association for Klotho. No significant association was found for FGF-23, and findings for urinary NGAL and KIM-1 were inconsistent across studies. Because of between-study differences, several biomarkers could not be pooled, and pooled analyses were limited to two studies. All findings should therefore be considered exploratory and require validation in larger prospective studies using standardized assays and outcome definitions.

## Data Availability

The original contributions presented in the study are included in the article/**Supplementary Material**. Further inquiries can be directed to the corresponding author.
