# Supplementary material for: Prognostic value of multiple biomarkers for kidney function decline in type 2 diabetes with relatively preserved kidney function: a systematic review and exploratory meta-analysis
Source: Front Endocrinol (Lausanne). 2026 Oct 12;17:1938521. doi: 10.3389/fendo.2026.1938521 (PMC13696286; doi:10.3389/fendo.2026.1938521)
Supplement: Supplementary file 1 [file DataSheet1.zip › Supplementary tables/Supplementary Table 1.docx]

| **Pubmed** | |
| --- | --- |
| # | Query |
| 1 | ((((((((((((((((((((((((((((((("Diabetes Mellitus, Type 2"[Mesh]) OR (Diabetes Mellitus, Stable[Title/Abstract])) OR (Stable Diabetes Mellitus[Title/Abstract])) OR (Diabetes Mellitus, Noninsulin Dependent[Title/Abstract])) OR (Diabetes Mellitus, Adult-Onset[Title/Abstract])) OR (Adult-Onset Diabetes Mellitus[Title/Abstract])) OR (Diabetes Mellitus, Adult Onset[Title/Abstract])) OR (Diabetes Mellitus, Ketosis-Resistant[Title/Abstract])) OR (Diabetes Mellitus, Ketosis Resistant[Title/Abstract])) OR (Ketosis-Resistant Diabetes Mellitus[Title/Abstract])) OR (Diabetes Mellitus, Non Insulin Dependent[Title/Abstract])) OR (Diabetes Mellitus, Non-Insulin-Dependent[Title/Abstract])) OR (Non-Insulin-Dependent Diabetes Mellitus[Title/Abstract])) OR (Diabetes Mellitus, Type II[Title/Abstract])) OR (NIDDM[Title/Abstract])) OR (Diabetes Mellitus, Maturity-Onset[Title/Abstract])) OR (Diabetes Mellitus, Maturity Onset[Title/Abstract])) OR (Maturity-Onset Diabetes Mellitus[Title/Abstract])) OR (Maturity Onset Diabetes Mellitus[Title/Abstract])) OR (MODY[Title/Abstract])) OR (Diabetes Mellitus, Slow-Onset[Title/Abstract])) OR (Diabetes Mellitus, Slow Onset[Title/Abstract])) OR (Slow-Onset Diabetes Mellitus[Title/Abstract])) OR (Type 2 Diabetes Mellitus[Title/Abstract])) OR (Noninsulin-Dependent Diabetes Mellitus[Title/Abstract])) OR (Noninsulin Dependent Diabetes Mellitus[Title/Abstract])) OR (Maturity-Onset Diabetes[Title/Abstract])) OR (Diabetes, Maturity-Onset[Title/Abstract])) OR (Maturity Onset Diabetes[Title/Abstract])) OR (Type 2 Diabetes[Title/Abstract])) OR (Diabetes, Type 2[Title/Abstract])) OR (Diabetes Mellitus, Noninsulin-Dependent[Title/Abstract]) |
| 2 | ((((((((((((((((("Diabetic Nephropathies"[Mesh]) OR (Nephropathies, Diabetic[Title/Abstract])) OR (Nephropathy, Diabetic[Title/Abstract])) OR (Diabetic Kidney Disease[Title/Abstract])) OR (Diabetic Kidney Diseases[Title/Abstract])) OR (Kidney Disease, Diabetic[Title/Abstract])) OR (Kidney Diseases, Diabetic[Title/Abstract])) OR (Diabetic Nephropathy[Title/Abstract])) OR (Diabetic Glomerulosclerosis[Title/Abstract])) OR (Glomerulosclerosis, Diabetic[Title/Abstract])) OR (Intracapillary Glomerulosclerosis[Title/Abstract])) OR (Kimmelstiel-Wilson Disease[Title/Abstract])) OR (Kimmelstiel Wilson Disease[Title/Abstract])) OR (Nodular Glomerulosclerosis[Title/Abstract])) OR (Glomerulosclerosis, Nodular[Title/Abstract])) OR (Kimmelstiel-Wilson Syndrome[Title/Abstract])) OR (Kimmelstiel Wilson Syndrome[Title/Abstract])) OR (Syndrome, Kimmelstiel-Wilson[Title/Abstract]) |
| 3 | #1 AND 2# |
| 4 | (((((((((((((((((("Receptors, Tumor Necrosis Factor, Type I"[Mesh]) OR (CD120a Antigen[Title/Abstract])) OR (Antigen, CD120a[Title/Abstract])) OR (TNFR p60[Title/Abstract])) OR (TNFR-I[Title/Abstract])) OR (Tumor Necrosis Factor Receptor Type I[Title/Abstract])) OR (TNFR1[Title/Abstract])) OR (Tumor Necrosis Factor Receptor 1A[Title/Abstract])) OR (TNFRSF1A (Tumor Necrosis Factor Receptor Superfamily, Member 1A[Title/Abstract]))) OR (Tumor Necrosis Factor Receptor Type 1[Title/Abstract])) OR (Antigens, CD120a[Title/Abstract])) OR (CD120a Antigens[Title/Abstract])) OR (TNFRSF1A Receptor[Title/Abstract])) OR (Receptor, TNFRSF1A[Title/Abstract])) OR (Tumor Necrosis Factor Receptor Superfamily, Member 1A[Title/Abstract])) OR (CD 120a Antigen[Title/Abstract])) OR (120a Antigen, CD[Title/Abstract])) OR (Antigen, CD 120a[Title/Abstract])) OR (Receptors, Tumor Necrosis Factor, Member 1A[Title/Abstract]) |
| 5 | ((((((((((((((((((((((("Receptors, Tumor Necrosis Factor, Type II"[Mesh])) OR (CD 120b Antigen[Title/Abstract])) OR (120b Antigen, CD[Title/Abstract])) OR (Antigen, CD 120b[Title/Abstract])) OR (CD120b Antigen[Title/Abstract])) OR (Antigen, CD120b[Title/Abstract])) OR (TNF-R75[Title/Abstract])) OR (TNF-RII[Title/Abstract])) OR (TNFR p75[Title/Abstract])) OR (Tumor Necrosis Factor Receptor 75[Title/Abstract])) OR (Tumor Necrosis Factor Receptor 2[Title/Abstract])) OR (TNFR2[Title/Abstract])) OR (Tumor Necrosis Factor Receptor Type 2[Title/Abstract])) OR (Tumor Necrosis Factor Receptor Type II[Title/Abstract])) OR (Antigens, CD120b[Title/Abstract])) OR (CD120b Antigens[Title/Abstract])) OR (TNFRSF1B Receptor[Title/Abstract])) OR (Receptor, TNFRSF1B[Title/Abstract])) OR (Tumor Necrosis Factor Receptor Superfamily, Member 1B[Title/Abstract])) OR (TNF-R2[Title/Abstract])) OR (Receptors, Tumor Necrosis Factor, Member 1B[Title/Abstract])) OR (TNF-sR75[Title/Abstract])) OR (TNFR p80[Title/Abstract]) |
| 6 | ("Fibroblast Growth Factor-23"[Mesh]) OR (Fibroblast Growth Factor 23[Title/Abstract]) |
| 7 | (((("Klotho Proteins"[Mesh]) OR (alpha-Klotho Proteins[Title/Abstract])) OR (alpha Klotho Proteins[Title/Abstract])) OR (beta-Klotho Proteins[Title/Abstract])) OR (beta Klotho Proteins[Title/Abstract]) |
| 8 | ((((((((("Hepatitis A Virus Cellular Receptor 1"[Mesh]) OR (HAVCR1 Protein[Title/Abstract])) OR (T-Cell Immunoglobulin[Title/Abstract])) OR (Mucin Domain-Containing Protein 1[Title/Abstract])) OR (T Cell Immunoglobulin[Title/Abstract])) OR (Mucin Domain Containing Protein 1[Title/Abstract])) OR (TIMD1 Protein[Title/Abstract])) OR (CD365 Antigen[Title/Abstract])) OR (Antigen, CD365[Title/Abstract])) OR (KIM-1[Title/Abstract]) |
| 9 | (((((((("Lipocalin-2"[Mesh]) OR (Lipocalin 2[Title/Abstract])) OR (Neutrophil Gelatinase-Associated Lipocalin[Title/Abstract])) OR (Neutrophil Gelatinase Associated Lipocalin[Title/Abstract])) OR (Lipocalin-2 Protein[Title/Abstract])) OR (Lipocalin 2 Protein[Title/Abstract])) OR (Oncogene 24p3 Protein[Title/Abstract])) OR (Siderocalin Protein[Title/Abstract])) OR (NGAL Protein[Title/Abstract]) |
| 10 | (((((#4) OR (#5)) OR (#6)) OR (#7)) OR (#8)) OR (#9) |
| 11 | #3 AND #10 |
| 12 | ((((((((((((((((((((((((((((((((("Cohort Studies"[Mesh] ) OR (Cohort Study[Title/Abstract])) OR (Studies, Cohort[Title/Abstract])) OR (Study, Cohort[Title/Abstract])) OR (Studies, Concurrent[Title/Abstract])) OR (Concurrent Study[Title/Abstract])) OR (Study, Concurrent[Title/Abstract])) OR (Concurrent Studies[Title/Abstract])) OR (Closed Cohort Studies[Title/Abstract])) OR (Cohort Studies, Closed[Title/Abstract])) OR (Closed Cohort Study[Title/Abstract])) OR (Cohort Study, Closed[Title/Abstract])) OR (Study, Closed Cohort[Title/Abstract])) OR (Studies, Closed Cohort[Title/Abstract])) OR (Historical Cohort Studies[Title/Abstract])) OR (Cohort Studies, Historical[Title/Abstract])) OR (Cohort Study, Historical[Title/Abstract])) OR (Historical Cohort Study[Title/Abstract])) OR (Study, Historical Cohort[Title/Abstract])) OR (Studies, Historical Cohort[Title/Abstract])) OR (Incidence Studies[Title/Abstract])) OR (Incidence Study[Title/Abstract])) OR (Studies, Incidence[Title/Abstract])) OR (Study, Incidence[Title/Abstract])) OR (Analysis, Cohort[Title/Abstract])) OR (Analyses, Cohort[Title/Abstract])) OR (Cohort Analyses[Title/Abstract])) OR (Cohort Analysis[Title/Abstract])) OR (Birth Cohort Studies[Title/Abstract])) OR (Birth Cohort Study[Title/Abstract])) OR (Cohort Studies, Birth[Title/Abstract])) OR (Cohort Study, Birth[Title/Abstract])) OR (Studies, Birth Cohort[Title/Abstract])) OR (Study, Birth Cohort[Title/Abstract]) |
| 13 | ((((((((((((((((("Longitudinal Studies"[Mesh]) OR (Longitudinal Study[Title/Abstract])) OR (Studies, Longitudinal[Title/Abstract])) OR (Study, Longitudinal[Title/Abstract])) OR (Longitudinal Survey[Title/Abstract])) OR (Longitudinal Surveys[Title/Abstract])) OR (Survey, Longitudinal[Title/Abstract])) OR (Surveys, Longitudinal[Title/Abstract])) OR (California Teachers Study[Title/Abstract])) OR (California Teachers Studies[Title/Abstract])) OR (Studies, California Teachers[Title/Abstract])) OR (Study, California Teachers[Title/Abstract])) OR (Teachers Studies, California[Title/Abstract])) OR (Teachers Study, California[Title/Abstract])) OR (Tuskegee Syphilis Study[Title/Abstract])) OR (Syphilis Studies, Tuskegee[Title/Abstract])) OR (Syphilis Study, Tuskegee[Title/Abstract])) OR (Tuskegee Syphilis Studies[Title/Abstract]) |
| 14 | ((("Prospective Studies"[Mesh]) OR (Prospective Study[Title/Abstract])) OR (Studies, Prospective[Title/Abstract])) OR (Study, Prospective[Title/Abstract]) |
| 15 | #12 OR #13 OR #14 |
| 16 | #11 AND #15 |

| **Cochrane Library** | |
| --- | --- |
| # | Query |
| 1 | MeSH descriptor: [Diabetes Mellitus, Type 2] explode all trees |
| 2 | (Diabetes Mellitus, Stable):ti,ab,kw OR (Stable Diabetes Mellitus):ti,ab,kw OR (Diabetes Mellitus, Noninsulin Dependent):ti,ab,kw OR (Diabetes Mellitus, Adult-Onset):ti,ab,kw OR (Adult-Onset Diabetes Mellitus):ti,ab,kw OR (Diabetes Mellitus, Adult Onset):ti,ab,kw OR (Diabetes Mellitus, Ketosis-Resistant):ti,ab,kw OR (Diabetes Mellitus, Ketosis Resistant):ti,ab,kw OR (Ketosis-Resistant Diabetes Mellitus):ti,ab,kw OR (Diabetes Mellitus, Non Insulin Dependent):ti,ab,kw OR (Diabetes Mellitus, Non-Insulin-Dependent):ti,ab,kw OR (Non-Insulin-Dependent Diabetes Mellitus):ti,ab,kw OR (Diabetes Mellitus, Type II):ti,ab,kw OR (NIDDM):ti,ab,kw OR (Diabetes Mellitus, Maturity-Onset):ti,ab,kw OR (Diabetes Mellitus, Maturity Onset):ti,ab,kw OR (Maturity-Onset Diabetes Mellitus):ti,ab,kw OR (Maturity Onset Diabetes Mellitus):ti,ab,kw OR (MODY):ti,ab,kw OR (Diabetes Mellitus, Slow-Onset):ti,ab,kw OR (Diabetes Mellitus, Slow Onset):ti,ab,kw OR (Slow-Onset Diabetes Mellitus):ti,ab,kw OR (Type 2 Diabetes Mellitus):ti,ab,kw OR (Noninsulin-Dependent Diabetes Mellitus):ti,ab,kw OR (Noninsulin Dependent Diabetes Mellitus):ti,ab,kw OR (Maturity-Onset Diabetes):ti,ab,kw OR (Diabetes, Maturity-Onset):ti,ab,kw OR (Maturity Onset Diabetes):ti,ab,kw OR (Type 2 Diabetes):ti,ab,kw OR (Diabetes, Type 2):ti,ab,kw OR (Diabetes Mellitus, Noninsulin-Dependent):ti,ab,kw |
| 3 | #1 OR #2 |
| 4 | MeSH descriptor: [Diabetic Nephropathies] explode all trees |
| 5 | (Nephropathies, Diabetic):ti,ab,kw OR (Nephropathy, Diabetic):ti,ab,kw OR (Diabetic Kidney Disease):ti,ab,kw OR (Diabetic Kidney Diseases):ti,ab,kw OR (Kidney Disease, Diabetic):ti,ab,kw OR (Kidney Diseases, Diabetic):ti,ab,kw OR (Diabetic Nephropathy):ti,ab,kw OR (Diabetic Glomerulosclerosis):ti,ab,kw OR (Glomerulosclerosis, Diabetic):ti,ab,kw OR (Intracapillary Glomerulosclerosis):ti,ab,kw OR (Kimmelstiel-Wilson Disease):ti,ab,kw OR (Kimmelstiel Wilson Disease):ti,ab,kw OR (Nodular Glomerulosclerosis):ti,ab,kw OR (Glomerulosclerosis, Nodular):ti,ab,kw OR (Kimmelstiel-Wilson Syndrome):ti,ab,kw OR (Kimmelstiel Wilson Syndrome):ti,ab,kw OR (Syndrome, Kimmelstiel-Wilson):ti,ab,kw |
| 6 | #4 OR #5 |
| 7 | #3 AND #6 |
| 8 | MeSH descriptor: [Receptors, Tumor Necrosis Factor, Type I] explode all trees |
| 9 | (CD120a Antigen):ti,ab,kw OR (Antigen, CD120a):ti,ab,kw OR (TNFR p60):ti,ab,kw OR (TNFR-I):ti,ab,kw OR (Tumor Necrosis Factor Receptor Type I):ti,ab,kw OR (TNFR1):ti,ab,kw OR (Tumor Necrosis Factor Receptor 1A):ti,ab,kw OR (TNFRSF1A (Tumor Necrosis Factor Receptor Superfamily, Member 1A):ti,ab,kw) OR (Tumor Necrosis Factor Receptor Type 1):ti,ab,kw OR (Antigens, CD120a):ti,ab,kw OR (CD120a Antigens):ti,ab,kw OR (TNFRSF1A Receptor):ti,ab,kw OR (Receptor, TNFRSF1A):ti,ab,kw OR (Tumor Necrosis Factor Receptor Superfamily, Member 1A):ti,ab,kw OR (CD 120a Antigen):ti,ab,kw OR (120a Antigen, CD):ti,ab,kw OR (Antigen, CD 120a):ti,ab,kw OR (Receptors, Tumor Necrosis Factor, Member 1A):ti,ab,kw |
| 10 | #8 OR #9 |
| 11 | MeSH descriptor: [Receptors, Tumor Necrosis Factor, Type II] explode all trees |
| 12 | (CD 120b Antigen):ti,ab,kw OR (120b Antigen, CD):ti,ab,kw OR (Antigen, CD 120b):ti,ab,kw OR (CD120b Antigen):ti,ab,kw OR (Antigen, CD120b):ti,ab,kw OR (TNF-R75):ti,ab,kw OR (TNF-RII):ti,ab,kw OR (TNFR p75):ti,ab,kw OR (Tumor Necrosis Factor Receptor 75):ti,ab,kw OR (Tumor Necrosis Factor Receptor 2):ti,ab,kw OR (TNFR2):ti,ab,kw OR (Tumor Necrosis Factor Receptor Type 2):ti,ab,kw OR (Tumor Necrosis Factor Receptor Type II):ti,ab,kw OR (Antigens, CD120b):ti,ab,kw OR (CD120b Antigens):ti,ab,kw OR (TNFRSF1B Receptor):ti,ab,kw OR (Receptor, TNFRSF1B):ti,ab,kw OR (Tumor Necrosis Factor Receptor Superfamily, Member 1B):ti,ab,kw OR (TNF-R2):ti,ab,kw OR (Receptors, Tumor Necrosis Factor, Member 1B):ti,ab,kw OR (TNF-sR75):ti,ab,kw OR (TNFR p80):ti,ab,kw |
| 13 | #11 OR #12 |
| 14 | MeSH descriptor: [Fibroblast Growth Factor-23] explode all trees |
| 15 | (Fibroblast Growth Factor 23):ti,ab,kw |
| 16 | #14 OR #15 |
| 17 | MeSH descriptor: [Klotho Proteins] explode all trees |
| 18 | (alpha-Klotho Proteins):ti,ab,kw OR (alpha Klotho Proteins):ti,ab,kw OR (beta-Klotho Proteins):ti,ab,kw OR (beta Klotho Proteins):ti,ab,kw |
| 19 | #17 OR #18 |
| 20 | MeSH descriptor: [Hepatitis A Virus Cellular Receptor 1] explode all trees |
| 21 | (HAVCR1 Protein):ti,ab,kw OR (T-Cell Immunoglobulin):ti,ab,kw OR (Mucin Domain-Containing Protein 1):ti,ab,kw OR (T Cell Immunoglobulin):ti,ab,kw OR (Mucin Domain Containing Protein 1):ti,ab,kw OR (TIMD1 Protein):ti,ab,kw OR (CD365 Antigen):ti,ab,kw OR (Antigen, CD365):ti,ab,kw OR (KIM-1):ti,ab,kw |
| 22 | #20 OR #21 |
| 23 | MeSH descriptor: [Lipocalin-2] explode all trees |
| 24 | (Lipocalin 2):ti,ab,kw OR (Neutrophil Gelatinase-Associated Lipocalin):ti,ab,kw OR (Neutrophil Gelatinase Associated Lipocalin):ti,ab,kw OR (Lipocalin-2 Protein):ti,ab,kw OR (Lipocalin 2 Protein):ti,ab,kw OR (Oncogene 24p3 Protein):ti,ab,kw OR (Siderocalin Protein):ti,ab,kw OR (NGAL Protein):ti,ab,kw |
| 25 | #23 OR #24 |
| 26 | #10 OR #13 OR #16 OR #19 OR #22 OR #25 |
| 27 | #7 AND #26 |
| 28 | MeSH descriptor: [Cohort Studies] explode all trees |
| 29 | (Cohort Study):ti,ab,kw OR (Studies, Cohort):ti,ab,kw OR (Study, Cohort):ti,ab,kw OR (Studies, Concurrent):ti,ab,kw OR (Concurrent Study):ti,ab,kw OR (Study, Concurrent):ti,ab,kw OR (Concurrent Studies):ti,ab,kw OR (Closed Cohort Studies):ti,ab,kw OR (Cohort Studies, Closed):ti,ab,kw OR (Closed Cohort Study):ti,ab,kw OR (Cohort Study, Closed):ti,ab,kw OR (Study, Closed Cohort):ti,ab,kw OR (Studies, Closed Cohort):ti,ab,kw OR (Historical Cohort Studies):ti,ab,kw OR (Cohort Studies, Historical):ti,ab,kw OR (Cohort Study, Historical):ti,ab,kw OR (Historical Cohort Study):ti,ab,kw OR (Study, Historical Cohort):ti,ab,kw OR (Studies, Historical Cohort):ti,ab,kw OR (Incidence Studies):ti,ab,kw OR (Incidence Study):ti,ab,kw OR (Studies, Incidence):ti,ab,kw OR (Study, Incidence):ti,ab,kw OR (Analysis, Cohort):ti,ab,kw OR (Analyses, Cohort):ti,ab,kw OR (Cohort Analyses):ti,ab,kw OR (Cohort Analysis):ti,ab,kw OR (Birth Cohort Studies):ti,ab,kw OR (Birth Cohort Study):ti,ab,kw OR (Cohort Studies, Birth):ti,ab,kw OR (Cohort Study, Birth):ti,ab,kw OR (Studies, Birth Cohort):ti,ab,kw OR (Study, Birth Cohort):ti,ab,kw |
| 30 | #28 OR #29 |
| 31 | MeSH descriptor: [Longitudinal Studies] explode all trees |
| 32 | (Longitudinal Study):ti,ab,kw OR (Studies, Longitudinal):ti,ab,kw OR (Study, Longitudinal):ti,ab,kw OR (Longitudinal Survey):ti,ab,kw OR (Longitudinal Surveys):ti,ab,kw OR (Survey, Longitudinal):ti,ab,kw OR (Surveys, Longitudinal):ti,ab,kw OR (California Teachers Study):ti,ab,kw OR (California Teachers Studies):ti,ab,kw OR (Studies, California Teachers):ti,ab,kw OR (Study, California Teachers):ti,ab,kw OR (Teachers Studies, California):ti,ab,kw OR (Teachers Study, California):ti,ab,kw OR (Tuskegee Syphilis Study):ti,ab,kw OR (Syphilis Studies, Tuskegee):ti,ab,kw OR (Syphilis Study, Tuskegee):ti,ab,kw OR (Tuskegee Syphilis Studies):ti,ab,kw |
| 33 | #31 OR #32 |
| 34 | MeSH descriptor: [Prospective Studies] explode all trees |
| 35 | (Prospective Study):ti,ab,kw OR (Studies, Prospective):ti,ab,kw OR (Study, Prospective):ti,ab,kw |
| 36 | #34 OR #35 |
| 37 | #30 OR #33 OR #36 |
| 38 | #27 AND #37 |

| **Web of Science** | |
| --- | --- |
| # | Query |
| 1 | (((((((((((((((((((((((((((((((TS=(Diabetes Mellitus, Type 2)) OR TS=(Diabetes Mellitus, Stable)) OR TS=(Stable Diabetes Mellitus)) OR TS=(Diabetes Mellitus, Noninsulin Dependent)) OR TS=(Diabetes Mellitus, Adult-Onset)) OR TS=(Adult-Onset Diabetes Mellitus)) OR TS=(Diabetes Mellitus, Adult Onset)) OR TS=(Diabetes Mellitus, Ketosis-Resistant)) OR TS=(Diabetes Mellitus, Ketosis Resistant)) OR TS=(Ketosis-Resistant Diabetes Mellitus)) OR TS=(Diabetes Mellitus, Non Insulin Dependent)) OR TS=(Diabetes Mellitus, Non-Insulin-Dependent)) OR TS=(Non-Insulin-Dependent Diabetes Mellitus)) OR TS=(Diabetes Mellitus, Type II)) OR TS=(NIDDM)) OR TS=(Diabetes Mellitus, Maturity-Onset)) OR TS=(Diabetes Mellitus, Maturity Onset)) OR TS=(Maturity-Onset Diabetes Mellitus)) OR TS=(Maturity Onset Diabetes Mellitus)) OR TS=(MODY)) OR TS=(Diabetes Mellitus, Slow-Onset)) OR TS=(Diabetes Mellitus, Slow Onset)) OR TS=(Slow-Onset Diabetes Mellitus)) OR TS=(Type 2 Diabetes Mellitus)) OR TS=(Noninsulin-Dependent Diabetes Mellitus)) OR TS=(Noninsulin Dependent Diabetes Mellitus)) OR TS=(Maturity-Onset Diabetes)) OR TS=(Diabetes, Maturity-Onset)) OR TS=(Maturity Onset Diabetes)) OR TS=(Type 2 Diabetes)) OR TS=(Diabetes, Type 2)) OR TS=(Diabetes Mellitus, Noninsulin-Dependent) |
| 2 | (((((((((((((((((TS=(Diabetic Nephropathies)) OR TS=(Nephropathies, Diabetic)) OR TS=(Nephropathy, Diabetic)) OR TS=(Diabetic Kidney Disease)) OR TS=(Diabetic Kidney Diseases)) OR TS=(Kidney Disease, Diabetic)) OR TS=(Kidney Diseases, Diabetic)) OR TS=(Diabetic Nephropathy)) OR TS=(Diabetic Glomerulosclerosis)) OR TS=(Glomerulosclerosis, Diabetic)) OR TS=(Intracapillary Glomerulosclerosis)) OR TS=(Kimmelstiel-Wilson Disease)) OR TS=(Kimmelstiel Wilson Disease)) OR TS=(Nodular Glomerulosclerosis)) OR TS=(Glomerulosclerosis, Nodular)) OR TS=(Kimmelstiel-Wilson Syndrome)) OR TS=(Kimmelstiel Wilson Syndrome)) OR TS=(Syndrome, Kimmelstiel-Wilson) |
| 3 | #1 AND #2 |
| 4 | ((((((((((((((((((TS=(Receptors, Tumor Necrosis Factor, Type I)) OR TS=(CD120a Antigen)) OR TS=(Antigen, CD120a)) OR TS=(TNFR p60)) OR TS=(TNFR-I)) OR TS=(Tumor Necrosis Factor Receptor Type I)) OR TS=(TNFR1)) OR TS=(Tumor Necrosis Factor Receptor 1A)) OR TS=(TNFRSF1A (Tumor Necrosis Factor Receptor Superfamily, Member 1A))) OR TS=(Tumor Necrosis Factor Receptor Type 1)) OR TS=(Antigens, CD120a)) OR TS=(CD120a Antigens)) OR TS=(TNFRSF1A Receptor)) OR TS=(Receptor, TNFRSF1A)) OR TS=(Tumor Necrosis Factor Receptor Superfamily, Member 1A)) OR TS=(CD 120a Antigen)) OR TS=(120a Antigen, CD)) OR TS=(Antigen, CD 120a)) OR TS=(Receptors, Tumor Necrosis Factor, Member 1A) |
| 5 | ((((((((((((((((((((((TS=(Receptors, Tumor Necrosis Factor, Type II)) OR TS=(CD 120b Antigen)) OR TS=(120b Antigen, CD)) OR TS=(Antigen, CD 120b)) OR TS=(CD120b Antigen)) OR TS=(Antigen, CD120b)) OR TS=(TNF-R75)) OR TS=(TNF-RII)) OR TS=(TNFR p75)) OR TS=(Tumor Necrosis Factor Receptor 75)) OR TS=(Tumor Necrosis Factor Receptor 2)) OR TS=(TNFR2)) OR TS=(Tumor Necrosis Factor Receptor Type 2)) OR TS=(Tumor Necrosis Factor Receptor Type II)) OR TS=(Antigens, CD120b)) OR TS=(CD120b Antigens)) OR TS=(TNFRSF1B Receptor)) OR TS=(Receptor, TNFRSF1B)) OR TS=(Tumor Necrosis Factor Receptor Superfamily, Member 1B)) OR TS=(TNF-R2)) OR TS=(Receptors, Tumor Necrosis Factor, Member 1B)) OR TS=(TNF-sR75)) OR TS=(TNFR p80) |
| 6 | (TS=(Fibroblast Growth Factor-23)) OR TS=(Fibroblast Growth Factor 23) |
| 7 | ((((TS=(Klotho Proteins)) OR TS=(alpha-Klotho Proteins)) OR TS=(alpha Klotho Proteins)) OR TS=(beta-Klotho Proteins)) OR TS=(beta Klotho Proteins) |
| 8 | (((((((((TS=(Hepatitis A Virus Cellular Receptor 1)) OR TS=(HAVCR1 Protein)) OR TS=(T-Cell Immunoglobulin)) OR TS=(Mucin Domain-Containing Protein 1)) OR TS=(T Cell Immunoglobulin)) OR TS=(Mucin Domain Containing Protein 1)) OR TS=(TIMD1 Protein)) OR TS=(CD365 Antigen)) OR TS=(Antigen, CD365)) OR TS=(KIM-1) |
| 9 | ((((((((TS=(Lipocalin-2)) OR TS=(Lipocalin 2)) OR TS=(Neutrophil Gelatinase-Associated Lipocalin)) OR TS=(Neutrophil Gelatinase Associated Lipocalin)) OR TS=(Lipocalin-2 Protein)) OR TS=(Lipocalin 2 Protein)) OR TS=(Oncogene 24p3 Protein)) OR TS=(Siderocalin Protein)) OR TS=(NGAL Protein) |
| 10 | #4 OR #5 OR #6 OR #7 OR #8 OR #9 |
| 11 | #3 AND #10 |
| 12 | (((((((((((((((((((((((((((((((((TS=(Cohort Studies )) OR TS=(Cohort Study)) OR TS=(Studies, Cohort)) OR TS=(Study, Cohort)) OR TS=(Studies, Concurrent)) OR TS=(Concurrent Study)) OR TS=(Study, Concurrent)) OR TS=(Concurrent Studies)) OR TS=(Closed Cohort Studies)) OR TS=(Cohort Studies, Closed)) OR TS=(Closed Cohort Study)) OR TS=(Cohort Study, Closed)) OR TS=(Study, Closed Cohort)) OR TS=(Studies, Closed Cohort)) OR TS=(Historical Cohort Studies)) OR TS=(Cohort Studies, Historical)) OR TS=(Cohort Study, Historical)) OR TS=(Historical Cohort Study)) OR TS=(Study, Historical Cohort)) OR TS=(Studies, Historical Cohort)) OR TS=(Incidence Studies)) OR TS=(Incidence Study)) OR TS=(Studies, Incidence)) OR TS=(Study, Incidence)) OR TS=(Analysis, Cohort)) OR TS=(Analyses, Cohort)) OR TS=(Cohort Analyses)) OR TS=(Cohort Analysis)) OR TS=(Birth Cohort Studies)) OR TS=(Birth Cohort Study)) OR TS=(Cohort Studies, Birth)) OR TS=(Cohort Study, Birth)) OR TS=(Studies, Birth Cohort)) OR TS=(Study, Birth Cohort) |
| 13 | (((((((((((((((((TS=(Longitudinal Studies)) OR TS=(Longitudinal Study)) OR TS=(Studies, Longitudinal)) OR TS=(Study, Longitudinal)) OR TS=(Longitudinal Survey)) OR TS=(Longitudinal Surveys)) OR TS=(Survey, Longitudinal)) OR TS=(Surveys, Longitudinal)) OR TS=(California Teachers Study)) OR TS=(California Teachers Studies)) OR TS=(Studies, California Teachers)) OR TS=(Study, California Teachers)) OR TS=(Teachers Studies, California)) OR TS=(Teachers Study, California)) OR TS=(Tuskegee Syphilis Study)) OR TS=(Syphilis Studies, Tuskegee)) OR TS=(Syphilis Study, Tuskegee)) OR TS=(Tuskegee Syphilis Studies) |
| 14 | (((TS=(Prospective Studies)) OR TS=(Prospective Study)) OR TS=(Studies, Prospective)) OR TS=(Study, Prospective) |
| 15 | #12 OR #13 OR #14 |
| 16 | #11 AND #15 |

| **Embase** | |
| --- | --- |
| # | Query |
| 1 | 'non insulin dependent diabetes mellitus'/exp AND [embase]/lim |
| 2 | ((((('adult onset diabetes'.ti,ab,kw OR 'adult onset diabetes mellitus':ti,ab,kw OR 'diabetes mellitus type 2':ti,ab,kw OR 'diabetes mellitus type ii':ti,ab,kw OR 'diabetes  Mellitus, maturity onset':ti,ab,kw OR 'diabetes mellitus, non insulin dependent':ti,ab,kw OR 'diabetes mellitus, non-insulin-dependent':ti,ab,kw OR 'diabetes mellitus,  type 2':ti,ab,kw OR 'diabetes mellitus, type ii':ti,ab,kw OR 'diabetes type 2':ti,ab,kw OR 'diabetes type ii':ti,ab,kw OR 'diabetes, adult onset':ti,ab,kw OR 'dm 2':ti,ab,kw OR  'insulin independent diabetes':ti,ab,kw OR 'insulin independent diabetes mellitus':ti,ab,kw OR 'ketosis resistant diabetes mellitus'ti,ab,kw OR 'maturity onset  diabetes':ti,ab,kw OR 'maturity onset diabetes mellitus':ti,ab,kw OR 'niddm':ti,ab,kw OR niddm:ti,ab,kw) AND 'non insulin dependent diabetes mellitus':ti,ab,kw OR 'non  insulin dependent':ti,ab,kw) AND 'type 2':ti,ab,kw AND 'diabetes mellitus':ti,ab,kw OR 'non insulin dependent diabetes':ti,ab,kw OR 'non-insulin-dependent diabetes  mellitus':ti,ab,kw OR 'noninsulin dependent':ti,ab,kw) AND 'type 2':ti,ab,kw AND 'diabetes mellitus':ti,ab,kw OR 'noninsulin dependent diabetes':ti,ab,kw OR 'noninsulin  dependent diabetes mellitus':ti,ab,kw OR 't2dm':ti,ab,kw OR 'tidm':ti,ab,kw OR 'type 2':ti,ab,kw) AND 'insulin independent':ti,ab,kw AND diabetes:ti,ab,kw OR 'type 2  diabetes':ti,ab,kw OR 'type 2 diabetes mellitus':ti,ab,kw OR 'type ii diabetes':ti,ab,kw OR 'type i diabetes mellitus':ti,ab,kw OR 'non insulin dependent diabetes  mellitus':ti,ab,kw) AND [embase]/lim |
| 3 | 'diabetic nephropathy'/exp AND [embase]/lim |
| 4 | ('diabetes nephropathy':ti,ab,kw OR 'diabetic kidney disease':ti,ab,kw OR 'diabetic nephropathies':ti,ab,kw OR 'diabetic renal disease':ti,ab,kw OR 'diabetic  nephropathy':ti,ab,kw) AND [embase]/lim |
| 5 | #1 OR #2 |
| 6 | #3 OR #4 |
| 7 | #5 AND #6 |
| 8 | 'tumor necrosis factor receptor 1'/exp AND [embase]/lim |
| 9 | ('cd120a antigen':ti,ab,kw OR 'receptors, tumor necrosis factor type i':ti,ab,kw OR 'receptors, tumour necrosis factor type i':ti,ab,kw OR 'tumor necrosis factor receptor type 1':ti,ab,kw OR 'tumor necrosis factor receptor type i':ti,ab,kw OR 'tumour necrosis factor receptor 1':ti,ab,kw OR 'tumour necrosis factor receptor type 1':ti,ab,kw OR 'tumour necrosis factor receptor type i':ti,ab,kw OR 'tumor necrosis factor receptor 1':ti,ab,kw) AND [embase]/lim |
| 10 | #8 OR #9 |
| 11 | 'tumor necrosis factor receptor 2'/exp AND [embase]/lim |
| 12 | ('cd120b antigen':ti,ab,kw OR 'receptors, tumor necrosis factor type ii':ti,ab,kw OR 'receptors, tumour necrosis factor type ii':ti,ab,kw OR 'tumor necrosis factor receptor type 2':ti,ab,kw OR 'tumor necrosis factor receptor type ii':ti,ab,kw OR 'tumour necrosis factor receptor 2':ti,ab,kw OR 'tumour necrosis factor receptor type 2':ti,ab,kw OR 'tumour necrosis factor receptor type ii':ti,ab,kw OR 'tumor necrosis factor receptor 2':ti,ab,kw) AND [embase]/lim |
| 13 | #11 OR #12 |
| 14 | 'klotho protein'/exp AND [embase]/lim |
| 15 | ('klotho proteins':ti,ab,kw OR 'protein klotho':ti,ab,kw OR 'klotho protein':ti,ab,kw) AND [embase]/lim |
| 16 | #14 OR #15 |
| 17 | 'fibroblast growth factor 23'/exp AND [embase]/lim |
| 18 | ('fgf 23':ti,ab,kw OR 'fgf23':ti,ab,kw OR 'fibroblast growth factor-23':ti,ab,kw OR 'phosphatonin':ti,ab,kw OR 'tumor derived hypophosphatemia factor':ti,ab,kw OR 'tumor  derived hypophosphatemia inducing factor':ti,ab,kw OR 'tumour derived hypophosphataemia factor':ti,ab,kw OR 'tumour derived hypophosphataemia inducing  factor':ti,ab,kw OR 'fibroblast growth factor 23':ti,ab,kw) AND [embase]/lim |
| 19 | #17 OR #18 |
| 20 | 'hepatitis a virus cellular receptor 1'/exp AND [embase]/lim |
| 21 | ('havcr 1 protein':ti,ab,kw OR 'havcr1 protein':ti,ab,kw OR 'kidney injury molecule 1':ti,ab,kw OR 'protein havcr 1':ti,ab,kw OR 'protein havcr1':ti,ab,kw OR 'protein tim 1':ti,ab,kw OR 'protein tim1':ti,ab,kw OR 'protein timd 1':ti,ab,kw OR 'protein timd1':ti,ab,kw OR 't cell immunoglobulin':ti,ab,kw OR 'mucin domain containing protein 1':ti,ab,kw OR 't-cell immunoglobulin':ti,ab,kw OR 'tim 1 protein':ti,ab,kw OR ''tim1 protein':ti,ab,kw OR 'timd 1 protein':ti,ab,kw OR 'timd1 protein':ti,ab,kw OR 'hepatitis a virus cellular receptor 1':ti,ab,kw) AND [embase]/lim |
| 22 | #20 OR #21 |
| 23 | 'neutrophil gelatinase associated lipocalin'/exp AND [embase]/lim |
| 24 | ('24p3 protein':ti,ab,kw OR 'lcn2 protein':ti,ab,kw OR 'lipocalin 2':ti,ab,kw OR 'lipocalin 24p3':ti,ab,kw OR 'lipocalin-2':ti,ab,kw OR 'ngal':ti,ab,kw OR 'ngal protein':ti,ab,kw OR 'protein 24p3':ti,ab,kw OR 'protein Icn2':ti,ab,kw OR 'protein ngal':ti,ab,kw OR 'protein sip24':ti,ab,kw OR 'siderochalin':ti,ab,kw OR 'sip24':ti,ab,kw OR 'sip24 protein':ti,ab,kw OR 'superinducible protein 24':ti,ab,kw OR 'uterocalin':ti,ab,kw OR 'neutrophil gelatinase associated ilpocalin':ti,ab,kw) AND [embase]/lim |
| 25 | #23 OR #24 |
| 26 | #10 OR #13 OR #16 OR #19 OR #22 OR #25 |
| 27 | 'cohort analysis'/exp AND [embase]/lim |
| 28 | ('analysis, cohort':ti,ab,kw OR 'cohort fertility':ti,ab,kw OR 'cohort life cycle':ti,ab,kw OR 'cohort studies':ti,ab,kw OR 'cohort study':ti,ab,kw OR 'fertility, cohort':ti,ab,kw  OR 'cohort analysis':ti,ab,kw) AND [embase]/lim |
| 29 | #27 OR #28 |
| 30 | #7 AND #26 AND #29 |
