# Supplementary material for: Prognostic value of multiple biomarkers for kidney function decline in type 2 diabetes with relatively preserved kidney function: a systematic review and exploratory meta-analysis
Source: Front Endocrinol (Lausanne). 2026 Oct 12;17:1938521. doi: 10.3389/fendo.2026.1938521 (PMC13696286; doi:10.3389/fendo.2026.1938521)
Supplement: Supplementary file 1 [file DataSheet1.zip › Supplementary tables/Supplementary Table 2.docx]

**Supplementary Table 2. GRADE certainty of evidence for each biomarker and kidney function decline.**

| **Review question** | **Body of evidence** | **Certainty** | **Reasons for downgrading** |
| --- | --- | --- | --- |
| In adults with type 2 diabetes and relatively preserved kidney function, is higher circulating TNFR1 associated with an increased risk of kidney function decline? | 4 prospective cohorts; pooled in two 2-study exploratory analyses (HR and OR) | Very low | Risk of bias; indirectness |
| In adults with type 2 diabetes and relatively preserved kidney function, is higher circulating TNFR2 associated with an increased risk of kidney function decline? | 2 prospective cohorts; pooled HR 1.82 (1.40–2.38) | Very low | Risk of bias; indirectness; imprecision |
| In adults with type 2 diabetes and relatively preserved kidney function, is lower circulating Klotho associated with an increased risk of kidney function decline? | 1 prospective cohort; single study | Very low | Risk of bias; imprecision |
| In adults with type 2 diabetes and relatively preserved kidney function, is higher circulating FGF-23 associated with kidney function decline? | 2 prospective cohorts; not pooled; both null | Very low | Risk of bias; indirectness; imprecision |
| In adults with type 2 diabetes and relatively preserved kidney function, is higher circulating KIM-1 associated with an increased risk of kidney function decline? | 3 prospective cohorts; 2 pooled (OR 1.38, 1.20–1.59); 1 not pooled | Very low | Risk of bias; inconsistency; indirectness; imprecision |
| In adults with type 2 diabetes and relatively preserved kidney function, is higher urinary KIM-1 associated with kidney function decline? | 3 prospective cohorts; not pooled; inconsistent directions | Very low | Risk of bias; inconsistency; indirectness; imprecision |
| In adults with type 2 diabetes and relatively preserved kidney function, is higher urinary NGAL associated with an increased risk of kidney function decline? | 2 prospective cohorts; not pooled; one significant, one not | Very low | Risk of bia; indirectness; imprecision |

GRADE certainty was initially set at moderate because all included studies were Phase 1 exploratory prognostic-factor studies. All biomarkers were ultimately rated very low certainty. The main reasons were risk of bias (most studies did not address competing risk of death), indirectness (mixed effect measures, exposure scales, and outcome definitions), and imprecision (very few studies per biomarker, ranging from 1 to 4). Publication bias could not be assessed because fewer than 10 studies were available (k<10) and was therefore not used for downgrading. Very low = we have very little confidence in the effect estimate; the true effect is likely substantially different.
